# MCL Plays an Anti-Inflammatory Role in *Mycobacterium tuberculosis*-Induced Immune Response by Inhibiting NF-*κ*B and NLRP3 Inflammasome Activation

**DOI:** 10.1155/2017/2432904

**Published:** 2017-05-31

**Authors:** Qingwen Zhang, Xinru Jiang, Weigang He, Kailin Wei, Jinxia Sun, Xiangyang Qin, Yuejuan Zheng, Xin Jiang

**Affiliations:** ^1^Department of Immunology and Microbiology, Shanghai University of Traditional Chinese Medicine, Shanghai, 201203, China; ^2^Major in Biology, The University of British Columbia, Vancouver, Canada V6T 1Z4; ^3^Department of Chemistry, School of Pharmacy, Fourth Military Medical University, Xi'an, Shaanxi 710034, China

## Abstract

*Mycobacterium tuberculosis* (Mtb) remains a significant menace to global health as it induces granulomatous lung lesions and systemic inflammatory responses during active tuberculosis (TB). Micheliolide (MCL), a sesquiterpene lactone, was recently reported to have a function of relieving LPS-induced inflammatory response, but the regulative role of MCL on the immunopathology of TB still remains unknown. In this experiment, we examined the inhibitory effect of MCL on Mtb-induced inflammatory response in mouse macrophage-like cell line Raw264.7 by downregulating the activation of nuclear factor kappa B (NF-*κ*B) and NLRP3 inflammasome. Evidences showed that MCL decreased the secretion of Mtb-induced inflammatory cytokines (IL-1*β* and TNF-*α*) in a dose-dependent manner. Meanwhile, MCL dramatically suppressed Mtb-induced activation of iNOS and COX2 as well as subsequent production of NO. Furthermore, MCL inhibited Mtb-induced phosphorylation of Akt (Ser 473) in Raw264.7. According to our results, MCL plays an important role in modulating Mtb-induced inflammatory response through PI3K/Akt/NF-*κ*B pathway and subsequently downregulating the activation of NLRP3 inflammasome. Therefore, MCL may represent as a potential drug candidate in the adjuvant treatment of TB by regulating host immune response.

## 1. Introduction

Host and pathogenic organisms are evolved in a complex and intensive cross-talk which results in the initiation of immune response. Inflammatory response plays a crucial protective role against various detrimental stimuli (e.g., Mtb). A potentially beneficial inflammation could become detrimental to organs once the inflammatory response becomes overwhelming [1, 2]. Excessive or improper production of inflammatory mediators, such as interleukin-1 (IL-1), tumor necrosis factor (TNF), and nitricoxide (NO), contributes to inflammatory disorder, tissue damage, organ failure, or death [2–5]. Especially in many severe TB, such as tuberculous meningitis or miliary TB, inflammatory response leads to harmful effects without any protection and requires additional anti-inflammatory therapy to suppress excessive inflammation [6]. For example, adjuvant therapy of TB with corticosteroids could reduce the mortality in patients with tuberculous meningitis [7, 8].

During TB, IL-1 is mainly produced in activated macrophages. The elevation of IL-1*β* could stimulate various immunological and inflammatory cells to synthesize proinflammatory cytokines, including TNF-*α*, IL-6, and NO, causing subsequently inflammatory and immunological damage [9, 10]. Studies have shown that production of elevated amounts of TNF-*α* caused severe inflammation in the lungs and spleen [11], and IL-1*β*-coated beads induced large granulomas in lung tissue [12]. Inflammasome is a hetero oligomeric multiprotein platform that activates caspase-1 and then facilitates the production of IL-1*β*. This complex consists of caspase-1 (the IL-1*β* processing enzyme), adapter protein ASC, and a sensor member of the NOD-like receptor (NLR) or AIM2-like receptor (ALR) families. Depending on NLR or ALR, inflammasome assembly and caspase-1 can be activated by either recognition of microbial patterns or through the detection of host cellular injury [13]. Consequently, IL-1*β* production is regulated through signal coordination derived from the existence of microbial colonization and the injury induced by the pathogen. It is noteworthy that the production of proinflammatory cytokines is controlled not only by NLR-related inflammasome but also by Toll-like receptor (TLR)-NF-*κ*B signaling. NF-*κ*B is a critical transcription factor downstream of TLR and participates in a broad spectrum of inflammatory diseases [14]. In a resting state, NF-*κ*B complex stays in the cytoplasm as a dimer consisting of p50 and p65 subunits which is inactivated by binding to inhibitor of NF-*κ*B (I*κ*B). In response to different proinflammatory stimuli, such as pathogens (i.e. Mtb), oxidative stress, and cytokines, I*κ*B is phosphorylated and degraded to release NF-*κ*B dimer, which is activated and translocated into nucleus, resulting in the transcription of proinflammatory factors, and so forth. Inducible nitric oxide synthase (iNOS) and cyclooxygenase 2 (COX2) are two downstream effectors of NF-*κ*B pathway [15] and are responsible for the production of NO and prostaglandin E2 (PGE2), respectively. Meanwhile, many studies also demonstrated that NF-*κ*B signaling pathway plays an essential role in NLRP3 inflammasome activation [16, 17]. IL-1*β* is transcribed and translated by NF-*κ*B as an inactive precursor (pro-IL-1*β*) and then is subsequently cleaved to its activated form (IL-1*β*) by NLRP3 inflammasome-activated caspase-1. In some circumstance (e.g., upon stimulation with insulin growth factor), phosphatidylinositol 3-kinase (PI3K)/Akt signaling pathway can induce the phosphorylation of IKK*α*/*β* and I*κ*B*α*, contributing to the activation of NF-*κ*B [18–20]. It is reported that deguelin (an Akt inhibitor) inhibited the activity of NF-*κ*B by inhibiting the phosphorylation and degration of I*κ*B*α* [18].

Tuberculosis (TB) remains to be the leading infectious cause of death worldwide, with 9.6 million new cases and 1.5 million deaths reported in 2014 [21]. The increasingly spread of multidrug-resistant TB (MDR-TB) and/or extensively drug-resistant TB (XDR-TB), HIV coinfection, and so forth, are the main barriers obstructing the treatment of TB nowadays. Furthermore, a disordered inflammation can cause a severe damage to the lung and other tissues characterized by an excessive secretion of inflammatory mediators (IL-1, TNF, NO, etc.) [22]. Of note, IL-1 can both initiate and aggravate the inflammation by recruiting leukocyte adhesion to the vascular endothelium and inducing the secondary production of chemokines which induce neutrophil chemotaxis to the damaged tissue [23]. Unlike other pathogen infections, the invaded Mtb could not be eradicated by inflammatory immune response completely. On the contrary, the excessive immune response causes significant damage to the host tissues [2, 24]. Most of the traditional anti-TB drugs aim to get rid of the pathogen and become less useful when Mtb gains drug-resistance. Actually, bacterial burden is not strictly correlated with disease progression [25]. The uncontrolled inflammation is the main etiology of severe TB [26, 27]. Thus, except for the development of new antibiotics, a new and promising approach to the treatment of TB has been focused, which as adjunctive therapy aims to limit Mtb infection and pathology by regulating the specific host immune pathways, namely host-directed therapies (HDTs) [28–30]. It contains two general approaches: modulating host inflammatory response to alleviate aberrant inflammation and protect the lung from severe destruction and enhancing the capacity of the host immune response against invading Mtb. Hence, it is important and feasible to look for new drugs suitable for HDTs from the abundant sources of natural herbs.

Sesquiterpene lactones (SLs) are a class of naturally occurring plant terpenoids which have more than 5000 compounds, and they have been reported harboring multiple biological activities, such as antiparasitic, antibacterial, antifungal, antiviral, anti-inflammatory, or immunomodulatory [31–33]. Micheliolide (MCL), a sesquiterpene lactone isolated from *Micheliacompressa* (Magnoliaceae), has been demonstrated to possess an anti-inflammatory effect on sepsis challenged by LPS [34]. Combining with the mechanisms involved in inflammatory signaling pathways, both NF-*κ*B pathway and NLRP3 inflammasome may serve as potential targets for the development of novel therapeutics to Mtb infection. In the current study, we demonstrated that MCL significantly inhibited the production of proinflammatory mediators (IL-1*β*, TNF-*α*, and NO) and played an inhibitory effect on Mtb-induced inflammatory response. The inhibition of Mtb-induced PI3K/Akt/NF-*κ*B signaling pathway and NLRP3 inflammasome by MCL may contribute to its anti-inflammatory role during Mtb infection.

## 2. Materials and Methods

### 2.1. Chemicals and Reagents

DMSO, LPS (0111:B4), and ATP were purchased from Sigma (St. Louis, MO). RIPA lysis buffer and Nitric Oxide Assay Kit were obtained from the Beyotime Institute of Biotechnology (Shanghai, China). Rabbit anti-NLRP3 monoclonal antibody was purchased from Cell Signaling Technology (Beverly, MA). Rabbit anti-ASC polyclonal antibody and rabbit anti-caspase-1 polyclonal antibody were from Santa Cruz Biotechnology Inc (Santa Cruz CA, USA), and mouse anti-*β*-actin monoclonal antibody was from Protein Tech Group (Chicago, IL). Micheliolide (MCL) (Molecular Weight: 248.3, purity >99%) was isolated from the *Michelia compressa* (Magnoliaceae) described previously [34]. Dulbecco's Modified Eagle's Medium (DMEM) and fetal bovine serum (FBS) (04-001-1A) were obtained from HyClone Laboratories Inc (Logan, UT, USA) and Biological Industries (BI, IL), respectively. Middle brook 7H9 media were obtained from Difco (Detroit, MI, USA), and oleic acid-albumin-dextrose-catalase (OADC) supplements were from BD Biosciences (BD, Sparks, MD, USA).

### 2.2. Cell Culture

The Raw264.7 murine macrophage cell line was cultured in DMEM supplemented with 10% FBS in 5% CO_2_ at 37°C.

### 2.3. Bacterial Strains

The Mtb H37Ra was used in this study. H37Ra strains were grown in Middle brook 7H9 or 7H11 broth supplemented with 0.2% glycerol, 0.05% Tween-80, and 10% Middle brook OADC supplement.

### 2.4. Mtb Infection

The Raw264.7 cells were seeded at a density of 1.0 × 10^6^ cells/well in a 6-well plate and grown at 37°C overnight. The cells were infected with Mtb H37Ra at a MOI (multiplicity of infection) of 10 : 1. After 4 hr of coincubation at 37°C, cells were washed three times with sterile PBS to remove extracellular bacteria and cultured with sterile PBS containing 10% FBS and different concentrations of MCL (5, 10, or 20 *μ*M) for different time courses.

### 2.5. Quantification of Proinflammatory Mediators

Four hours after the Mtb infection, the cells were washed 3 times with sterile PBS and replaced with fresh culture medium (contain 10% FBS). Meanwhile, different concentrations of MCL (5, 10, or 20 *μ*M) were added to the medium. The culture supernatants were collected, and the secretion of TNF-*α* and IL-1*β* was examined by enzyme-linked immunosorbent assay (ELISA) kits (R&D Systems, Minneapolis, MN) according to the manufacturer's instructions. Nitric oxide (NO) generation was assessed by Nitric Oxide Assay Kit.

### 2.6. RNA Quantification

Cells were lysed, and total RNA was extracted using Trizol reagent (Invitrogen, Carlsbad, CA). The complementary cDNA was transcripted from 1 *μ*g RNA using reverse transcriptase (Takara, Dalian, China). Quantitative real-time PCR was carried out using LightCycler (Roche Diagnostics, Indianapolis, IN) with SYBR Premix Ex Taq (Takara, Dalian, China). The mRNA level of GAPDH was measured as an internal control, and data were normalized with it.

### 2.7. Western Blot Analysis

Cells were collected and lysed in lysis buffer (Beyotime Institute of Biotechnology), and then the whole cell lysate was separated by SDS-PAGE and further transferred onto nitrocellulose membranes (Pall, USA). After blocking with TBST (0.5% Tween-20) containing 5% (*w*/*v*) nonfat milk, the membranes were incubated with specific primary antibodies against NLRP3 (1 : 1000), iNOS (1 : 500), COX2 (1 : 500), p65 (1 : 1000), or pp65 (1 : 1000) at 4°C overnight in blocking solution. Following 3 times of wash with TBST, the membranes were incubated with HRP-conjugated secondary antibodies (anti-rabbit and anti-mouse, resp.) at room temperature for 1 hr. The chemiluminescence was detected using the ECL-chemiluminescent kit (Thermo Scientific) with Protein Simple (USA).

### 2.8. Immunofluorescence

Following with the appropriate treatment, the cells were washed twice with PBS, fixed with 4% paraformaldehyde at room temperature for 10 min, and washed again with PBS. The cells were treated with penetrating reagents (consisted of 0.2% of BSA, 2% of TritonX-100) for 10 min at 4°C and washed again with PBS, then the cell were blocked with 5% bovine serum albumin (BSA, Sigma-Aldrich) for 30 min at room temperature. Rabbit anti-ASC (10500-1-AP, proteintech), anti-LC3 (sc-16755, Santa Cruz Biotechnology), anti-caspase-1 (sc-514, Santa Cruz Biotechnology), and anti-pp65 (cst-3033, cell signaling technology) antibodies were used for immunoblot. Donkey anti-mouse IgG (sc-2094, Santa Cruz Biotechnology) and goat anti-rabbit FITC conjugated antibodies (sc-2012, Santa Cruz Biotechnology) were used as secondary antibodies. The nuclei were stained with DAPI at the concentration of 1 *μ*g/ml for 10 min. In this experiment, confocal microscopy (LSM 880, Zeiss optics international trading co. LTD) was used to examine.

### 2.9. Assay of Luciferase Reporter Gene Expression

NF-*κ*B luciferase reporter assay was conducted previously [34] with the mixture of 100 ng plasmid, and 20 ng pRL-TK-Renilla luciferase plasmid was cotransfected into Raw264.7 cells. After 30 hr, cells were infected with H37Ra at MOI of 10 and treated with MCL (10 *μ*M) for 12 hr and the luciferase activities were measured according to the previous study [34].

### 2.10. Statistical Analysis

Statistical analysis was performed by using SPSS 18.0 (SPSS Inc., Chicago, IL, USA). *P* values were assessed using variance (ANOVA) one-way analysis, and results were given as the means ± standard deviation (SD). Data shown are representative of at least triplicate experiments. A value of *p* < 0.05 was considered to be statistically significant.

## 3. Results

### 3.1. MCL Decreases the Production of Proinflammatory Cytokines Induced by Mtb Infection in Raw264.7

**Figure 1:**
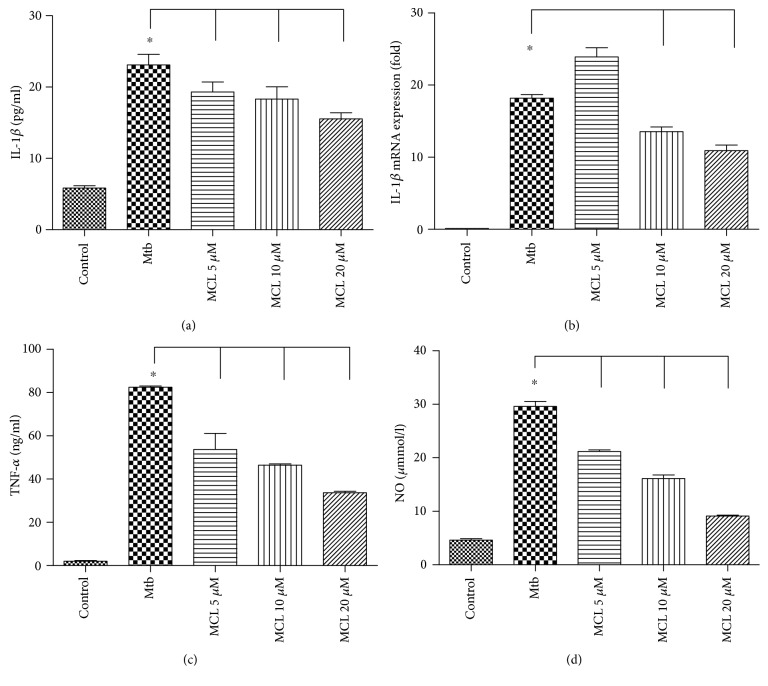
MCL inhibits Mtb-induced proinflammatory cytokine secretion. Raw264.7 cells (1 × 10^6^/well) were seeded in 6-well plates overnight and infected with H37Ra at a MOI of 10. Four hours after the infection, cells were washed with sterile PBS and then were treated with different concentrations of MCL (5, 10, and 20 *μ*M) for 12 hr. The concentrations of IL-1*β* (a) or TNF-*α* (c) in the supernatants were measured by ELISA, and mRNA expression of IL-1*β* (b) was detected by qRT-PCR. The concentration of NO (d) in the supernatants was measured with Nitric Oxide Assay Kit. Data are shown as the means ± SD (*n*≧3). ^∗^*p* < 0.05.

**Figure 2:**
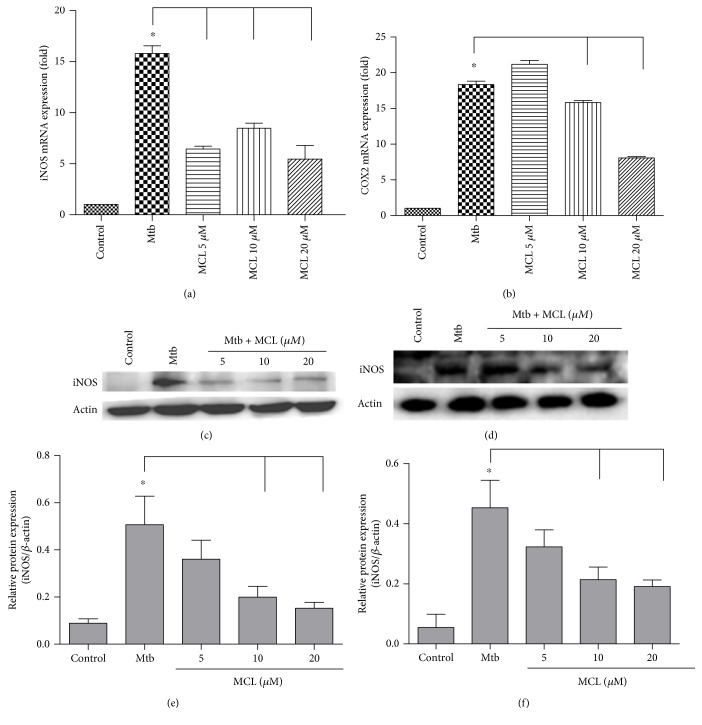
MCL suppresses the expression of iNOS and COX2 induced by Mtb infection both at transcriptional and at translational levels. Raw264.7 cells (1 × 10^6^/well) were seeded in 6-well plates overnight. Four hours after infected with H37Ra at MOI of 10, cells were washed with PBS and treated with different concentration of MCL (5, 10, and 20 *μ*M) for 12 hr. The mRNA expression of iNOS (a) and COX2 (b) was detected by qRT-PCR. Data are shown as the means ± SD (*n*≧3). ^∗^*p* < 0.05.The protein levels of iNOS (c) and COX2 (d) were detected by Western blot, and *β*-actin was used as an internal control. (e) and (f) showed the statistical results for iNOS and COX2 expression. Data were presented as mean ± SD in at least three independent experiments. ^∗^*p* < 0.05.

Macrophages serve as the major host cell niche for intracellular growth of different microorganisms including Mtb. As one of the principal antigen presenting cells, macrophages are also the inflammatory cytokine-producing cells which play an important role in the process of inflammatory diseases. The regulatory role of MCL on the inflammatory response was observed in Mtb-infected macrophages. Due to a cytotoxicity assay of MCL [34], the concentrations used in this study were safe for macrophages. As shown in Figures 1(a) and 1(b), MCL inhibited Mtb-induced IL-1*β* both at the transcriptional level and at translational level. Meanwhile, MCL inhibited the secretion of TNF-*α* and NO (Figures 1(c) and 1(d)). MCL also decreased the expression of iNOS and COX2 at transcriptional and translational levels (Figures 2(a), 2(b), 2(c), 2(d), 2(e), and 2(f)), which were responsible for NO and PGE2 production, respectively. Hence, we demonstrated that MCL can downregulate not only the secretion of IL-1*β* and TNF-*α*, but also attenuates the production of iNOS, accounting for the secretion of NO in Mtb-infected Raw264.7.

### 3.2. MCL Inhibits Mtb-Induced Activation of NLRP3 Inflammasome

**Figure 3:**
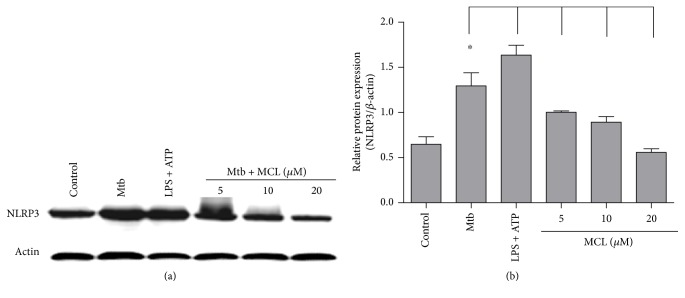
MCL inhibits Mtb-induced NLRP3 production. Raw264.7 cells (1 × 10^6^/well) were seeded in 6-well plates overnight and with H37Ra infection as mentioned before, and cells were then treated with MCL at the concentration (5, 10, and 20 *μ*M) for 12 hr. (a) The protein expression of NLRP3 was detected by Western blot, and *β*-actin was used as an internal control. (b) The bar graphs showed the statistical results for NLRP3 expression. Data are presented as mean ± SD in at least three independent experiments, ^∗^*p* < 0.05.

**Figure 4:**
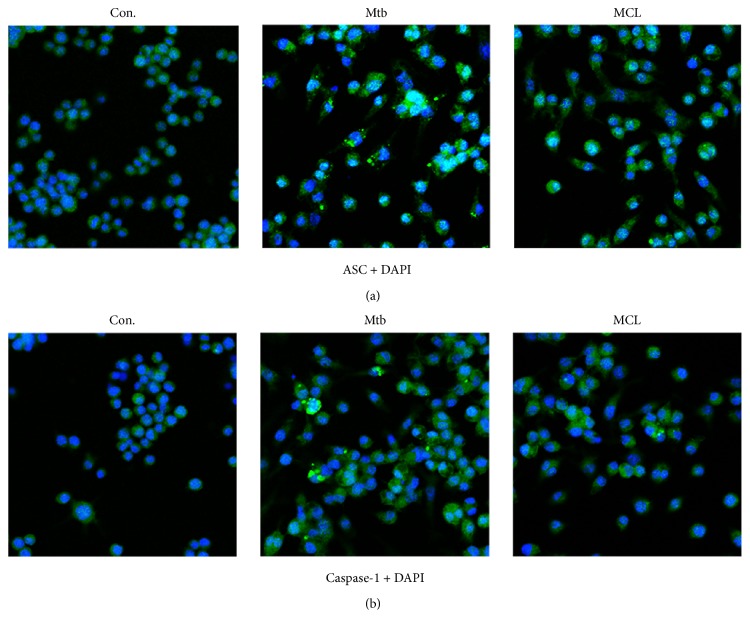
MCL attenuates the amount of NLRP3 inflammasome components in Mtb-infected Raw264.7 cell. Raw264.7 cells (1 × 10^5^/well) were seeded in 6-well plates which were precovered with cell climbing slice overnight, and the cells were infected with H37Ra as previously. Cells were then treated with MCL at the concentration of 10 *μ*M for 12 hr. Rabbit anti-ASC (green) or rabbit anti-caspase-1 (green) antibody and DAPI (blue) were used for immunostaining assay. Images were taken with laser scanning confocal microscopy. Con. is the abbreviation of control group, which showed no effect on the expression of ASC (a) and caspase-1 (b). Mtb promoted the formation and amounts of ASC specks, and the treatment of MCL reduced it apparently (a). Meanwhile, consistent with ASC, Mtb infection evidently augments the expression of caspase-1 and MCL decreased caspase-1 (b). Thus, MCL treatment apparently inhibits the activity of NLRP3 inflammasome. The results of (a) and (b) were obtained, respectively.

NLRP3 inflammasome acts as the molecular platform for the inactive cytoplasmic precursor (pro-IL-1*β*) processing into active IL-1*β*. We have observed the inhibitory effect of MCL on Mtb-induced IL-1*β*, so it is not difficult to speculate that Mtb infection is likely to induce the activation of NLRP3 inflammasome and MCL may disturb this process. To verify our hypothesis, the following Western blot and immunofluorescence assay were performed. As shown in Figure 3, MCL dramatically inhibited Mtb-induced NLRP3 protein expression. Meanwhile, MCL prevented the formation of ASC specks and the production of caspase-1 induced by Mtb infection (Figure 4). ASC and caspase-1 are the essential components of NLRP3 inflammasome assembly. So far, MCL may play an anti-inflammatory role by inhibiting the activation of NLRP3 inflammasome.

### 3.3. MCL Inhibits Mtb-Induced NF-*κ*B Activation

**Figure 5:**
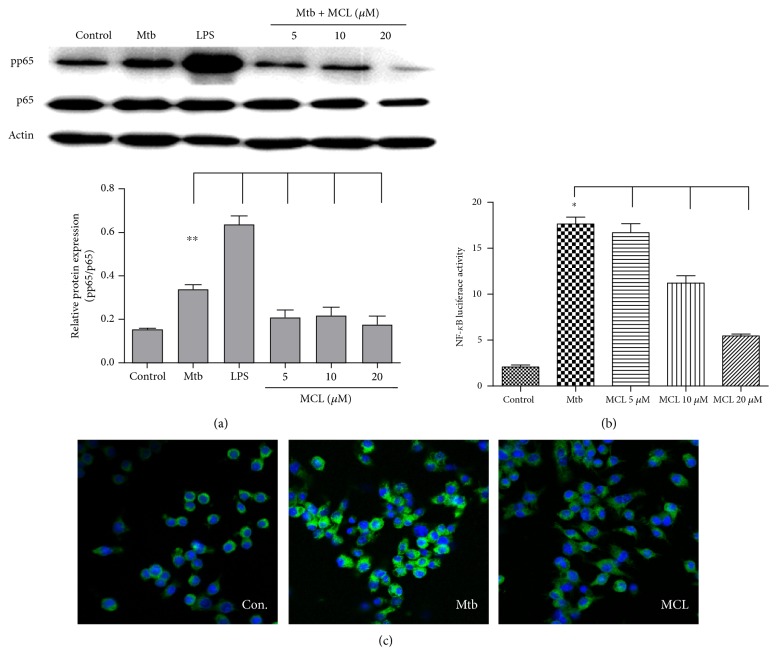
MCL inhibits Mtb-triggered NF-*κ*B activation. Raw264.7 cells (1 × 10^6^/well) were seeded in 6-well plates and infected with Mtb for 4 hrs. Cells were washed by PBS and treated with MCL (10 *μ*M) for indicated time courses. Phosphorylation of p65 and *β*-actin was detected by Western blot (a). The lower bar graph showed the statistical results for phosphorylation levels of NF-*κ*B p65 (a). Data are presented as mean ± SD in at least three independent experiments, ^∗∗^*p* < 0.01. Raw264.7 cells (3 × 10^5^/well) were cotransfected with NF-*κ*B luciferase reporter plasmid and pRL-TKR Renilla luciferase plasmid as previous study [34]. After 30 hr, cells were infected with H37Ra at MOI of 10 and treated with MCL (10 *μ*M) for 12 hr, and the luciferase activities were measured (b). Data are shown with the means ± SD (*n*≧3). ^∗^*p* < 0.05. Raw264.7cells (3 × 10^5^/well) were plated in climbing slice and followed by appropriate treatment. Immunofluorescence assay was conducted with rabbit anti-pp65 antibody (green) and DAPI (blue) (c). The results represented three independent experiments.

NF-*κ*B signaling pathway plays an essential role in the transcription of proinflammatory cytokines and contributes to the activation of NLRP3 inflammasome. We examined the effect of MCL on NF-*κ*B signaling pathway. MCL treatment inhibited the phosphorylation of p65 subunit of NF-*κ*B (Figure 5(a)), hindering the translocation of NF-*κ*B. Upon the stimulation of Mtb, a reporter gene assay was carried out to further clarify the regulatory effect of MCL on NF-*κ*B activation. LPS-treated cells were used as a positive control. As shown in Figure 5(b), Mtb treatment increased NF-*κ*B luciferase activity obviously, and MCL inhibited Mtb-induced NF-*κ*B activation in a dose-dependent manner. Consistent results were obtained in an immunofluorescence assay (Figure 5(c)). MCL restrained the translocation of NF-*κ*B to the nucleus. Therefore, MCL inhibits NF-*κ*B activation following Mtb infection.

### 3.4. MCL Inhibits PI3K/Akt Signaling Pathway

**Figure 6:**
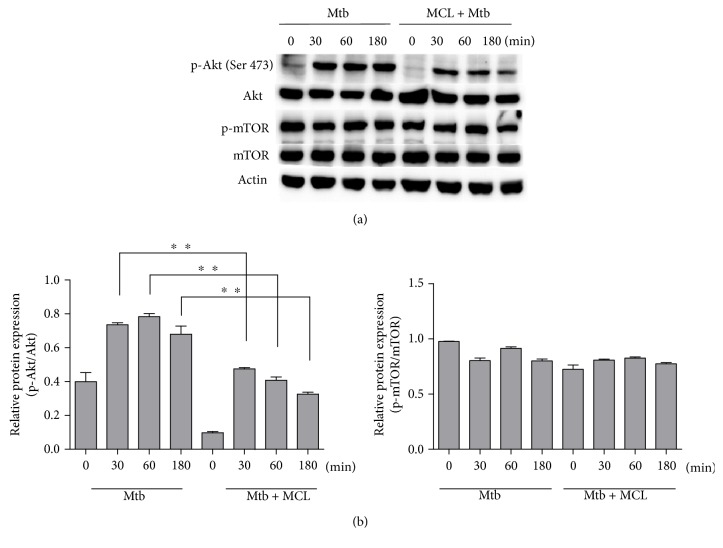
MCL inhibits the phosphorylation of Akt. Raw264.7 cells (1 × 10^6^/well) were seeded in 6-well plates overnight and subsequently infected with Mtb for 4 hr. After washing with PBS, cells were treated with or without MCL (10 *μ*M) for 30, 60 or 180 min. The protein level of Akt, p-Akt (Ser 473), mTOR, p-mTOR, and *β*-actin were detected by Western blot (a). The bar graphs showed the statistical results for phosphorylation levels of Akt and mTOR (b). Data are shown with the means ± SD (*n*≧3). ^∗∗^*p* < 0.01.

PI3K/Akt pathway involves in the progress of inflammation and influences NF-*κ*B activation as well [35, 36]. To investigate whether this pathway participates in the inhibitory effect of MCL on Mtb-induced immune response, we detected the phosphorylation of Akt and mTOR for different time courses upon MCL treatment. Results showed that MCL inhibited the phosphorylation of Akt (Ser 473) (Figure 6) but had no obvious effects on the phosphorylation of mTOR. MCL played a rapid inhibition of Akt phosphorylation upon Mtb stimulation, which indicated that MCL showed its regulatory function in the activation of PI3K/Akt signaling pathway at early phase.

### 3.5. MCL Has Little Effects on Autophagy Pathway

**Figure 7:**
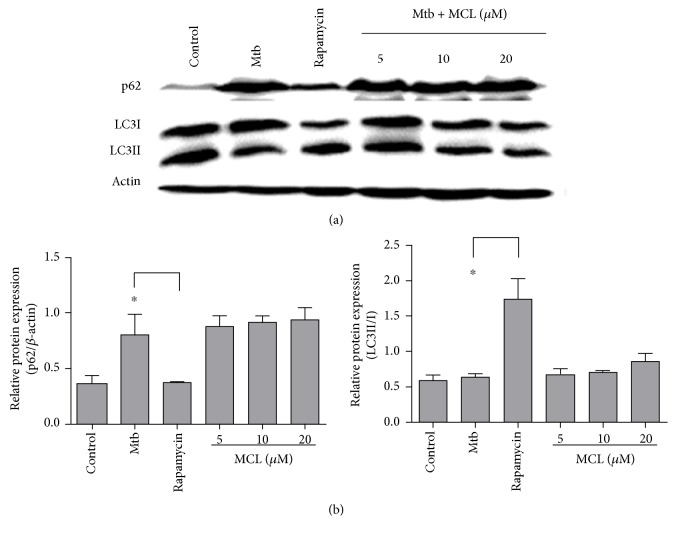
MCL has little impact on autophagy pathway. Raw264.7 cells (1 × 10^6^/well) were plated in 6-well plates overnight. Four hours after Mtb infection, cells were treated with or without MCL (10 *μ*M) or rapamycin (1 mg/ml) for 12 hr. The LC3I, LC3II, and p62 were detected by Western blot (a). The bar graphs showed the statistical results for the ratio of LC3II/LC3I and p62 expression (b). Data are shown with the means ± SD (*n*≧3). ^∗^*p* < 0.05.

It was reported that inflammasome and autophagosome play a mutual regulatory role of each other [37]. The induction of inflammasome can activate autophagy which in turn limits the activation of inflammasome through the degration of inflammasome-associated components. As shown in Figure 3, MCL inhibited Mtb-induced NLRP3 expression, downregulating the activation of NLRP3 inflammasome. Thus, we wondered whether MCL's downregulatory effect on NLRP3 inflammasome was associated with autophagy function. Autophagy-associated proteins LC3I/II and p62 which represent the activity of autophagy [38] were examined by Western blot. MCL treatment had rare effects on the activation of autophagy (Figure 7). Rapamycin, a known inhibitor of mTOR, can induce autophagy definitely [39, 40], so we choose it as the positive control. Compared with rapamycin treatment, MCL treatment did not decrease the level of p62 expression. In other words, MCL treatment did not alter the activation of autophagy and MCL exerts that its anti-inflammatory role does not depend on affecting autophagy pathways.

## 4. Discussion

Dysregulated inflammation represents a central pathogenic characteristic of multiple life-threatening microbial infections, including Mtb [2]. For millennia, TB (caused by Mtb infection) has been a principal threat and is still a big obstacle to public health [41]. Traditional anti-TB drugs are directed against microbial itself but cannot completely control a wide range of epidemic [21]. Instead of the conventional antimycobacterial therapies, more attention has been paid on the regulation of host immune response which is named host-directed therapies (HDTs) and might serve as adjunctive therapy. The goal of HDTs is either to directly augment the ability of the host immune system or to minimize collateral tissue damage caused by deleterious inflammation [28–30]. Inflammasome is an intracellular molecular platform which controls the production of IL-1 and is involved in the process of TB-related inflammatory damage. There are reports indicated that targeting on inflammasome activity may be one of the promising methods in the treatment of TB [30]. Our previous work showed that MCL plays an anti-inflammatory and protective role in LPS-induced sepsis [34]. In this study, we demonstrated that MCL is a potential drug candidate of TB by regulating the host inflammatory response.

Mtb is endowed with the unique ability to regulate fundamental inflammatory processes, such as recruiting immune cells to the focus of infection to produce proinflammatory cytokines (IL-1*β*, TNF-*α*, etc.). IL-1 is a critical proinflammatory cytokine which can initiate and amplify the inflammatory response through inflammatory cascade effects by inducing chemokines and subsequently neutrophil chemotaxis to the infection site. However, this defense mechanism also brings severe damage to the host tissue when attacking Mtb since the releasing hydrolytic enzymes and reactive oxygen species (ROS) produced by activated phagocytes are equally toxic to host and microbial cells [26]. Thus, in the context of persistent infections caused by Mtb infection, it is important to manage the potentially destructive aspects of the IL-1-initiated inflammatory response to prevent the host tissue from injuring. Recently, it was shown that controlling the production of mature IL-1*β* alleviates Mtb-induced host lung and spleen immunological injury [42]. In vitro studies showed that Mtb activates NLRP3 inflammasome and results in the production of mature IL-1*β* in infected macrophages [43, 44]. In this study, evidences were shown that MCL inhibited Mtb-induced NLRP3 inflammasome activation and the secretion of mature IL-1*β* in mouse macrophage-like cell line Raw264.7. As we know, TNF-*α* has long been considered as a vital inflammatory mediator which can cause severe injury to organs and even septic shock [45]. During TB, inappropriate production of TNF-*α* is detrimental to the host causing aggravated tissue pathology [46, 47]. Our data showed that MCL also plays an inhibitory role in Mtb-induced TNF-*α* secretion.

Nitric oxide (NO) is an important messenger, generated by a family of NO synthetases (NOSs) [48], and is involved in a variety of inflammatory diseases [49]. Overexpression of NO by macrophages has been implicated in bacterial septic shock [50] and certain inflammatory or autoimmune diseases [51]. Thus, suppression of the excessive NO production is a new therapeutic strategy for chronic inflammatory diseases [49]. Another inflammatory mediator, PGE2, is synthesized by cyclooxygenase 2 (COX2) and also participates in many inflammatory diseases (e.g., TB) [52]. Here, we demonstrated that Mtb-induced NO production, as well as iNOS and COX2, was dramatically restrained by MCL treatment.

NF-*κ*B, a ubiquitously existed transcription factor family, plays pleiotropic regulatory roles on the expression of more than 150 genes and is involved in a broad range of important life processes including inflammation, immunity, cell proliferation, and survival [53, 54]. To further explore the molecular mechanism of MCL, we investigated the activity of NF-*κ*B by phosphorylation of p65, its translocation to nucleus, and the transcriptional activity of NF-*κ*B. As expected, Mtb infection induced the activation of NF-*κ*B, while MCL treatment inhibited Mtb-induced NF-*κ*B activation in a dose-dependent manner.

Studies have shown that PI3K/Akt signaling pathway also contributes to the activation of NF-*κ*B [18, 19]. MCL inhibited Mtb-induced phosphorylation of Akt on Ser 473 from an early time point (30 min), and this inhibitory role lasted at least till the end of examination (3 hr). Such an inhibitory role on PI3K/Akt activation might influence the downstream NF-*κ*B activation [35, 36]. It suggested that MCL plays the anti-inflammatory role likely through PI3K/Akt/NF-*κ*B signaling pathway.

**Figure 8:**
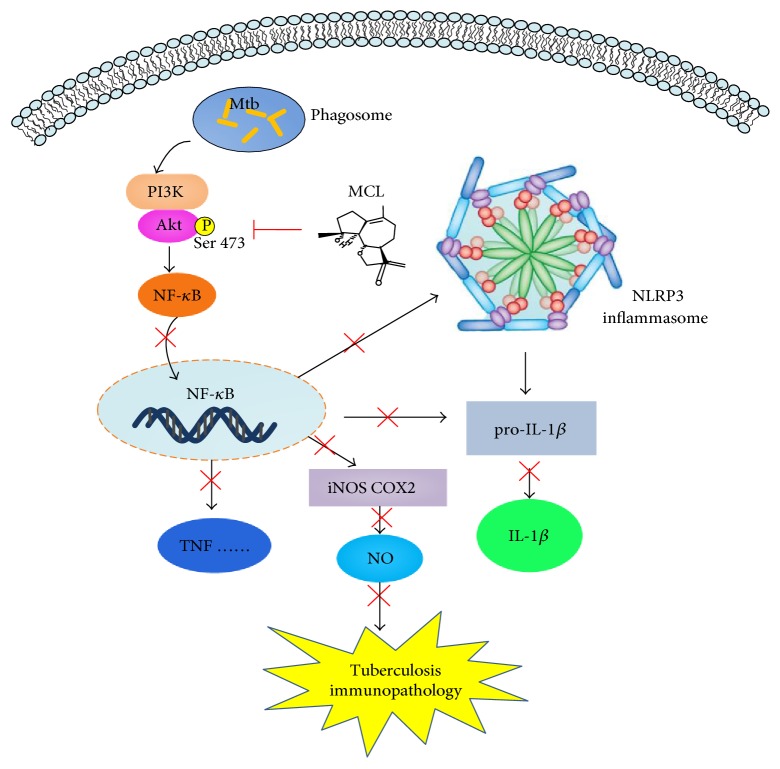
Illustration of the mechanism underlying the inhibitory effect of MCL on Mtb-induced immunopathologic response. MCL can suppress the phosphorylation of Akt causing the subsequently inhibitory effect on NF-*κ*B and NLRP3 inflammasome, which then impair the production of proinflammatory cytokines and finally alleviate tuberculosis immunopathology.

In our study, MCL is characterized as a potent adjuvant candidate which might be used to regulate Mtb-associated inflammation and relieve the relevant pathological damage of TB. The conclusions were based on the following observations: foremost, treatment with MCL at concentration of 5–20 *μ*M significantly inhibited the production of Mtb-induced proinflammatory factors in a dose-dependently way (Figures 1 and 2). Then, the activation of NF-*κ*B signaling pathway induced by Mtb was dramatically inhibited by MCL as proved via Western blot, dual-luciferase reporter assay and immunofluorescence (Figure 5), and early inhibitory effect on phosphorylated Akt (Figure 6). Last but not least, the production of NLRP3 was evidently reduced by MCL treatment (Figure 3), and at the same time, ASC and caspase-1, the essential components of NLRP3 inflammasome, were detected by immunofluorescence assay, data demonstrated that MCL remarkably inhibited the formation of ASC specks and the produce of caspase-1 (Figure 4). Taken together, MCL takes a crucial role in relieving Mtb-induced inflammatory reaction via modulating the activity of PI3K/Akt/NF-*κ*B signaling pathway and NLRP3 inflammasome (Figure 8). Our study brought a new perspective and platform of host-directed therapies (HDTs) to find potential drugs of anti-TB.
